# TASK-1 potassium channel is not critically involved in mediating hypoxic pulmonary vasoconstriction of murine intra-pulmonary arteries

**DOI:** 10.1371/journal.pone.0174071

**Published:** 2017-03-16

**Authors:** Ghulam Murtaza, Petra Mermer, Anna Goldenberg, Uwe Pfeil, Renate Paddenberg, Nobert Weissmann, Guenter Lochnit, Wolfgang Kummer

**Affiliations:** 1 Institute of Anatomy and Cell Biology, Justus-Liebig-University, Giessen, Germany; 2 Universities of Giessen and Marburg Lung Center, Justus-Liebig-University, Giessen, Germany; 3 German Center for Lung Research, Excellence Cluster Cardio-Pulmonary System, Justus-Liebig-University, Giessen, Germany; 4 Institute of Biochemistry, Faculty of Medicine, Justus-Liebig University, Giessen, Germany; Vanderbilt University Medical Center, UNITED STATES

## Abstract

The two-pore domain potassium channel KCNK3 (TASK-1) is expressed in rat and human pulmonary artery smooth muscle cells. There, it is associated with hypoxia-induced signalling, and its dysfunction is linked to pathogenesis of human pulmonary hypertension. We here aimed to determine its role in hypoxic pulmonary vasoconstriction (HPV) in the mouse, and hence the suitability of this model for further mechanistic investigations, using appropriate inhibitors and TASK-1 knockout (KO) mice. RT-PCR revealed expression of TASK-1 mRNA in murine lungs and pre-acinar pulmonary arteries. Protein localization by immunohistochemistry and western blot was unreliable since all antibodies produced labelling also in TASK-1 KO organs/tissues. HPV was investigated by videomorphometric analysis of intra- (inner diameter: 25–40 μm) and pre-acinar pulmonary arteries (inner diameter: 41–60 μm). HPV persisted in TASK-1 KO intra-acinar arteries. Pre-acinar arteries developed initial HPV, but the response faded earlier (after 30 min) in KO vessels. This HPV pattern was grossly mimicked by the TASK-1 inhibitor anandamide in wild-type vessels. Hypoxia-provoked rise in pulmonary arterial pressure (PAP) in isolated ventilated lungs was affected neither by TASK-1 gene deficiency nor by the TASK-1 inhibitor A293. TASK-1 is dispensable for initiating HPV of murine intra-pulmonary arteries, but participates in sustained HPV specifically in pre-acinar arteries. This does not translate into abnormal rise in PAP. While there is compelling evidence that TASK-1 is involved in the pathogenesis of pulmonary arterial hypertension in humans, the mouse does not appear to serve as a suitable model to study the underlying molecular mechanisms.

## Introduction

Alveolar hypoxia induces contraction of pulmonary arteries, a phenomenon known as hypoxic pulmonary vasoconstriction (HPV), resulting in redistribution of blood from poorly to optimally ventilated lung segments. Initially, HPV is a protective response but chronic hypoxia may lead to pulmonary hypertension (PH) [1]. The two-pore domain K^+^ channel, KCNK3 (potassium channel subfamily K member 3), also known as TASK-1 (TWIK-related acid-sensitive K^+^ channel-1), has been implicated both in molecular mechanisms of HPV and in pathogenesis of PH. It is acid sensitive and inhibited by anandamide [2] and A293 [3]. Closure of this channel decreases K^+^ efflux, causing membrane depolarization with subsequent opening of voltage-dependent Ca^2+^ channels and increase in intracellular Ca^2+^. Increased concentration of Ca^2+^ within smooth muscle cells (SMC) of vessels will cause vasoconstriction [4]. Carotid body glomus cells show marked hypoxia-sensitive TASK activity [5, 6] which is absent in TASK-1 knockout (KO) mice [7, 8], and these mice show a marked reduction of the hypoxia-evoked increase in carotid sinus nerve discharge [9]. These data point toward a contribution of TASK-1 in oxygen-dependent cellular signalling.

Accordingly, KCNK3 is expressed in oxygen-sensitive pulmonary arterial smooth muscle cells (PASMC) of rat [10], human [11], and rabbit [12], where it controls the resting membrane potential and is blocked by moderate hypoxia. Long-term (7–28 days) inhibition of this channel by A293 induces signs of PH in rats and elevated right ventricular systolic pressure [13]. KCNK3 expression and function are reduced in rat PASMC in monocrotaline-induced PH, and the KCNK3 activator ONO-RS-082 significantly ameliorates development of PH in this model [13]. In humans, *knck3* missense mutations have been identified in PH patients [14–16], and pulmonary KCNK3 expression and KCNK3 currents in PASMC are also diminished in PH patients who do not carry this mutation [13].

These data demonstrate that KCNK3 is causally involved in hypoxia-induced signalling in PASMC and in PH pathogenesis. The initial mechanisms triggering reduced KCNK3 expression in inflammatory PH models and linking hypoxia to KCNK3 inhibition, however, are poorly understood. This channel is not directly regulated by oxygen itself [17, 18] and may require associated proteins to serve as an oxygen sensor [18]. An established binding partner is KCNK9, also known as TASK-3, a member of the same K^+^ channel family [19], and forming heterodimers with TASK-1 in carotid body glomus cells [8] and motoneurons [20]. It may compensate for the absence of TASK-1 [21, 22]. However, there is also no evidence for its direct O_2_-sensitivity. Further elucidation of the underlying mechanisms would be facilitated by using appropriate genetically modified animal strains, preferably mice. First attempts to elucidate the role of KCNK3 and KCNK9 in the mouse pulmonary vasculature have focused upon first to third order intra-pulmonary arteries (0.1–0.5 mm in diameter). In TASK-1 and TASK-3 double (TASK1/3) KO mice, however, the constrictory responses recorded by myography and the electrophysiological properties of their PASMC were indistinguishable from those of wild-type (WT) mice, implying that TASK-1 does not form a functional channel in these arteries [23]. Accordingly, this particular segment of the murine pulmonary vascular tree, much in contrast to that of the rat, showed only small and inconsistent constrictory responses to hypoxia so that it is not a suitable model for studying mechanisms of HPV [23]. In mice, clear HPV can be monitored in the most distally located pre-acinar (41–100 μm in diameter) and intra-acinar (20–40 μm in diameter) pulmonary arteries by videomicroscopy of precision-cut lung slices (PCLS) [24, 25] and hypoxia-induced changes in pulmonary arterial pressure (PAP) can be recorded from isolated buffer-perfused and ventilated lungs [26]. Thus, we used these approaches to study the potential role of KCNK3 in HPV in the mouse lung, and hence the suitability of this model for further mechanistic investigations, using appropriate inhibitors and TASK-1 KO mice.

## Methods and materials

### Animals

All experiments were conducted according to the recommendations and directions on the use and care of experimental animals provided by National Institutes of Health and approved by the Regierungspräsidium Giessen, Germany (approval # GI 20/23, A7/2009 and GI 20/10 # A23/2008). Animals were 14–16 weeks old and belonged to both sexes. TASK-1 KO mice [21] used were of C57BL/6, WT, genetic background. For immunohistochemistry, western blot, and polymerase chain reaction (PCR) experiments, tissues were isolated from mice killed by an overdose of inhaled isoflurane and exsanguinated by cutting the abdominal vessels. For HPV study, mice were sacrificed by cervical dislocation. For PAP analysis, mice were deeply anesthetized with ketamine/xylazine and killed by exsanguination under anesthesia.

### DNA isolation and genotyping

Genotyping of TASK-1 mice was performed by PCR using genomic DNA extracted from either tail cuts or ear biopsies with the DNeasy Blood & Tissue Kit (Qiagen, Hilden/Germany) according to the manufacturer’s recommendations. PCR conditions: Initial activation at 95°C for 3 min, 5 cycles with 95°C for 30 s, 60°C for 20 s, 72°C for 30 s, and finally 35 cycles with 95°C for 30 s, 57°C for 20 s, and 72°C for 30 s. Primers used: 5’-TCATCGTGTGCAACCTTCACC-3’, 5’-CCTTCTATCGCCTTCTTGACG-3’, 5’-TGATGGCGAAGTAGAAGGAGC-3’. PCR products were separated on 2% Tris-acetate agarose gels. The expected amplicon sizes were 234 bp for WT and 344 bp for KO mice.

### RNA isolation, Reverse Transcription Polymerase Chain Reaction (RT-PCR)

Total RNA from the heart, lung, and cerebellum (at least n = 3 for each) was extracted using RNeasy Micro Kit (Qiagen, Hilden/Germany) according to the manufacturer’s protocol.

Complementary DNA (cDNA) synthesis: To remove any genomic DNA contamination, 1 μg RNA in 8 μl water was incubated with 1 μl 10x DNase reaction buffer and 1 μl DNase (1 U/μl; Invitrogen, Darmstadt/Germany) for 15 min at 25°C. Then, 1 μl ethylenediaminetetraacetic acid (25 mM) was added to each sample. After 10 min incubation at 65°C, samples were rapidly chilled on ice and 9 μl reaction mixture containing 1 μl oligo-dT (50 μM), 1 μl dNTPs (10 mM), 1 μl Superscript RNase H^-^ Reverse Transcriptase (RT; 200 U/μl), 4 μl 5x first-strand buffer, and 2 μl dithiothreitol (DTT; 0.1 M; all reagents were purchased from Invitrogen except dNTPs which were from Qiagen) was added to each sample.

PCR was performed with cDNA samples using following protocol: 4 μl cDNA as template, 2 μl MgCl_2_ (25 mM), 2.5 μl 10x PCR buffer II, 0.5 μl dNTPs (10 mM), 0.5 μl of each primer (10 μM), 0.2 μl AmpliTaq Gold DNA Polymerase (5 U/μl; all reagents were obtained from Applied Biosystems, Darmstadt/Germany), and 14.8 μl H_2_O. Cycling conditions: Initial denaturation at 95°C for 12 min, followed by 39 cycles of 20 s at 95°C, 20 s at 60°C, and 20 s at 72°C. Primers used in PCR: TASK-1 forward (F) 5’-CCTTCTACTTCGCCATCACC-3’, reverse (R) 5’-GACACGAAACCGATGAGCAC-3’, GenBank accession # NM010608, TASK-3 F 5’-CGCCCTCGAGTCGGACCATG-3’, R 5’-ACCAGCGTCAGGGGGATACCC-3’, GenBank accession # NM001033876, β-actin F 5’-GTGGGAATGGGTCAGAAGG-3’, R 5’-GGCATACAGGGACAGCACA-3’, GenBank accession # NM007393.

Laser-assisted microdissection and pressure-catapulting technology (PALM Microlaser Technologies, Bernried/Germany): Cardiomyocytes (*n* = 2), bronchi (*n* = 1), and SMC of intra- and pre-acinar arteries (*n* = 2 for each) were isolated from 6 μm thick cryosections of thoracic packages of WT mice. Each piece of microdissected tissue contained several cells. About 60 of such samples (pieces of tissue) for each specimen were picked and processed for RT-PCR. Methods of preparation, cell picking, total RNA extraction, and cDNA synthesis followed by qualitative PCR were as described in [27]. In our protocol before cell picking, samples collected on glass slides were also stained with eosin for 1–2 min. Excessive dye was removed through rinsing slides with slow running tap water for 1–2 min and then dipping in increasing concentrations of ethanol (70%, 80%, and 100%). Primers used in PCR are listed above. In PCR experiments, a positive control (cDNA of heart) that was successfully used in previous PCRs was included to check that the PCR conditions used could successfully amplify the target sequence. The absence of any contaminating genomic DNA was validated by including reactions without RT during the first round of cDNA synthesis. Samples were also processed with no template controls (H_2_O). Amplicons were examined on ethidium bromide-stained 2% agarose gels. The expected PCR product lengths were 250 bp for TASK-1, 288 bp for TASK-3, and 300 bp for β-actin.

### Immunohistochemistry

Lungs of WT and TASK-1 KO mice were removed, placed in cryo-embedding medium (Tissue-Tek, Sakura/Netherlands), and shock-frozen in liquid nitrogen. Specimens were sectioned at 10 μm thickness with a cryostat microtome (Leica CM 1900, Wetzlar/Germany) and mounted onto Superfrost Plus glass slides (R. Langenbrinck, Emmendingen/Germany). Sections were air-dried and fixed in acetone for 10 min at -20°C. Next, nonspecific antibody binding sites were blocked by incubating lung cryosections for 1 h in blocking solution consisting of (a) 5% bovine serum albumin (BSA), 5% normal goat serum in phosphate buffered saline (PBS), or (b) 10% normal swine serum, 0.5% Tween 20, 0.1% BSA in PBS. Samples were incubated overnight at room temperature with one of the following primary antibodies: (i) Polyclonal rabbit anti-TASK-1 antibody raised against a peptide corresponding to amino acid residues 252–269 of human TASK-1 (Cat # APC-024, lots # AN 02 and 06, Alomone Labs, Jerusalem/Israel), (ii) polyclonal rabbit anti-TASK-1 antibody from Chemicon (AB5250), (iii) two different polyclonal rabbit anti-TASK-1 antibodies (codes R-10/14 and R-10/15), self-made and pre-purified on Superdex-200 size exclusion chromatography columns, gifted by Prof. Rüdiger Veh, Charité—Universitätsmedizin Berlin/Germany. Anti-TASK-1 antibodies were applied in combination with monoclonal FITC-labelled anti-α-smooth muscle actin antibody (1:500, clone 1A4, Sigma Aldrich, Steinheim/Germany) to identify SMC. After washing in PBS, samples were incubated for 1 h at room temperature with Cy3-conjugated donkey anti-rabbit Ig (Millipore, Schwalmbach/Germany). Afterwards, samples were washed in PBS, post-fixed in 4% paraformaldehyde for 10 min, and finally washed in PBS at room temperature. Sections were mounted with a drop of Mowiol 4–88 (pH 8.6; Merck, Darmstadt/Germany) and examined under a Zeiss Axioplan 2 epifluorescence microscope (Jena/Germany) equipped with appropriate filter sets.

### Western blot

Preparation of extracts, sodium dodecyl sulphate polyacrylamide gel electrophoresis (SDS-PAGE), and western blotting were performed as previously described [24, 28]. Briefly, lung, heart, and cerebellum were isolated from TASK-1 WT, heterozygous (HZ), and KO mice. Organs or pieces of organs were weighed and the 5-fold volume of extraction buffer (10 mM Tris-HCl pH 6.8, 1% SDS, 10% glycerol, 7 M urea, 5 mM DTT, 0.5 mM phenylmethylsulfonylfluorid and 1x concentrated Complete Mini Protease Inhibitor Cocktail (Roche Diagnostics, Mannheim/Germany) was added. Tissues were homogenized by a ball mill (Mixer Mill MM300; Retsch GmbH, Haan/Germany) and total protein concentrations were measured by Bio-Rad Protein assay (Bio-Rad Laboratories, Munich/Germany). Thirty micrograms protein were loaded per lane, separated by 10% SDS-PAGE and transferred onto polyvinylidene fluoride membranes (PVDF; Millipore, Schwalbach/Germany). The membranes were incubated at room temperature for 1 h with blocking buffer (Tris-buffered saline (TBS), 0.05% Tween 20, 10% milk powder). Thereafter, membranes were incubated overnight at room temperature with different lots of primary polyclonal rabbit anti-TASK-1 antibody (Cat # APC-024, lots # AN 02 and AN 08; Alomone Labs) at 1:2,000 dilution in TBS, 0.05% Tween 20, 5% milk powder. The membranes were washed with TBS, 0.05% Tween 20 and then incubated for 1 h at room temperature with horseradish peroxidase-conjugated goat anti-rabbit IgG secondary antibody (Pierce/Perbio Science, Bonn/Germany) at 1:10,000 dilution in TBS, 0.05% Tween 20, and 2.5% milk powder. Bound antibodies were visualized using one volume of Super Signal West Dura Extended Duration Substrate (Pierce/Perbio Science) mixed with 9 volumes of Super Signal West Pico Chemiluminescent Substrate and X-ray film (Amersham/GE Healthcare Europe GmbH; Munich/Germany).

### Two-Dimensional (2-D) gel electrophoresis and western blotting

2-D Gel electrophoresis followed by western blotting was performed as described earlier [29, 30]. Briefly, cerebellum was dissected from WT and TASK-1 KO mice. Pieces of organs were solubilized in 6 M urea, 2 M thiourea, 4% 3-((3-cholamidopropyl) dimethyl ammonio)-1-propane sulfonate, 1% DTT, and 2% Pharmalyte. After rehydration of immobilized pH gradient (IPG) strips (pH 3–10 nonlinear; GE Healthcare, Freiburg/Germany) at 20°C, 250 μg proteins were applied on each strip and isoelectric focusing was performed with 32.05 kVh. Subsequently, the IPG strips were equilibrated for 10 min in 2 ml equilibration stock solution (ESS; 6 M urea, 0.1 mM ethylenediaminetetraacetic acid, 0.01% bromophenol blue, 50 mM Tris-HCl pH 6.8, 30% glycerol) for 15 min in 2 ml ESS I (10 ml ESS containing 200 mg SDS, 100 mg DTT) followed by 15 min in ESS II (10 ml ESS containing 200 mg SDS, 480 mg iodacetamide). Protein separation in the second dimension was performed by 10% SDS-PAGE. Electrophoresis was carried out in a Hoefer 600 system with the following program: 15 min at 15 mA/gel and 5 h at 110 mA at 25°C. Gels were stained with Flamingo (Bio-Rad Laboratories, Munich/Germany) and scanned with a Typhoon 9100 laser scanner (GE Healthcare). Densitometric analysis of the gels was done with PDQuest software (Bio-Rad Laboratories). Subsequently, proteins were transferred onto PVDF membranes using Towin-buffer for 16.3 h at 35 V and 570 Vh. Membranes were blocked with Roti-Block (Roth, Karlsruhe/Germany) for 3 h. Anti-TASK-1 antibody lot AN 02 was diluted in Roti-Block (2 μl antibody in 10 ml) and the membranes were incubated over night at 4°C. Horseradish peroxidase-conjugated swine anti-rabbit Ig, Dako P0217 (DakoCytomation, Hamburg/Germany), was used as secondary antibody (1:2,000 dilution in Roti-Block) for 1 h. For enhanced chemiluminescence (ECL) detection, the ECL Prime Western Blotting Detection Reagent Kit (GE Healthcare) was used.

Spots obtained on membranes were digested after reduction and carbamidomethylation with trypsin using an automated liquid handling system (MicroStarlet, Hamilton Robotics, Martinsried/Germany). Tryptic peptides were eluted from the gel plugs with 1% trifluoric acid. Matrix-assisted laser-desorption ionization time-of-flight mass spectrometry (MALDI-TOF-MS) was performed on an Ultraflex TOF/TOF mass spectrometer equipped with a nitrogen laser and a LIFT-MS/MS facility. The instrument was operated in the positive-ion reflectron mode using 2.5-dihydroxybenzoic acid and methylendiphosphonic acid as matrix. Sum spectra consisting of 200–400 single spectra were acquired. For data processing and instrument control the Compass 1.1 software package consisting of FlexControl 2.4, FlexAnalysis 3.0 and BioTools 3.0 was used. Proteins were identified by MASCOT peptide mass fingerprint search (http://www.matrixscience.com) using the mouse International Protein Index (IPI) database 20110927 (59534 sequences; 26627161 residues). For the search a mass tolerance of 75 ppm was allowed and carbamidomethylation of cysteine as global modification and oxidation of methionine as variable modification were used. A false positive rate of 5% was allowed.

### Videomorphometric analysis of intra-pulmonary arteries using PCLS

PCLS were prepared and analysed for vasoreactivity as described previously [25]. To investigate HPV, intra-pulmonary arteries were divided into intra-acinar arteries with lumen diameters 25–40 μm and pre-acinar arteries with lumen diameters 41–60 μm. The lumen diameter in section profiles was determined at 90° angle to the longest axis of the lumen. Effects of TASK-1 inhibitors anandamide (10 μM; Sigma-Aldrich, Steinheim/Germany) and A293 (200 nM; gift from Sanofi Aventis, Frankfurt/Germany) on pulmonary vasoconstriction under normoxic and hypoxic conditions were investigated in intra-pulmonary arteries. At the start of each experiment, viability of vessels was tested by exposing them to 0.1 μM U46619, a thromboxane analog (Sigma-Aldrich, Deisenhofen/Germany) and to 25 μM sodium nitroprusside (Nipruss, Schwarz Pharma GmbH Deutschland, Monheim/Germany). Only those vessels were considered for investigation, which responded to U46619, with at least 20% reduction of the lumen area. This initial testing phase was excluded from the graphs. Same amounts of solvents (ethanol for anandamide and dimethyl sulfoxide (DMSO) for A293) were added in control experiments.

### Lung isolation, perfusion, and ventilation

Hypoxia-induced changes in PAP were studied in WT and TASK-1 KO mice. Isolation, perfusion, and ventilation of mouse lungs were performed as described previously [26]. Briefly, mice were deeply anesthetized with ketamine (125 mg/kg body weight, i.p.) and xylazine (25 mg/kg body weight, i.p.) and treated with an i.p. application of anticoagulant heparin (2500 U/kg). Animals were subjected to intubation through a tracheostoma followed by ventilation with room air (positive pressure ventilation, 10 μl/g body weight tidal volume, 90 breaths/min and 2 cm H_2_O positive end expiratory pressure). Catheters were then inserted into the pulmonary artery and the left atrium. Perfusion with Krebs-Henseleit buffer (120 mM NaCl, 4.3 mM KCl, 1.1 mM KH_2_PO_4_, 2.4 mM CaCl_2_, 1.3 mM MgCl_2_, 13.3 mM glucose, 5% (w/v) hydroxyethylamylopectin (molecular weight 200,000); Serag-Wiessner, Naila/Germany) was performed via pulmonary artery at a flow rate of 0.2 ml/min and 4°C was commenced using peristaltic pump (ISM834A V2.10, Ismatec, Glattbrugg/Switzerland). With the inception of artificial perfusion, ventilation was continued with a gas mixture containing 21% O_2_, 5.3% CO_2_, balanced with N_2_. After initial rinsing of the lungs with 20 ml buffer the perfusion course was closed for subsequent recirculation (10 ml total system volume, time set to zero), and pressure of left atrium was adjusted at 2.0 mmHg. In the meantime, the flow was gradually increased to 2 ml/min and the temperature of the entire system was set at 37°C. Pressure in the pulmonary artery was recorded via catheters. After reaching a stable state, lungs were once ventilated with hypoxic gas (1% O_2_, 5.3% CO_2_, balanced with N_2_) for 10 min followed by ventilation with normoxic gas (21% O_2_, 5.3% CO_2_, balanced with N_2_) for 15 min. Afterwards, sustained hypoxic ventilation was performed for a period of 180 min. At the end of the 180 min period of hypoxic ventilation, lungs were ventilated with normoxic gas for 15 min and hypoxic gas medium for 10 min again followed by normoxic ventilation. Effects of 50 μM A293 on PAP under hypoxic conditions were also assessed in WT mice. Data are shown for the 180 min period of hypoxic ventilation as well as the subsequent 10 min hypoxic ventilation period.

### Statistical analysis

Data are presented as mean ± standard error of the mean (SEM). For videomorphometry data, analysis of differences among experimental groups was performed with the Kruskal-Wallis-test followed by the Mann–Whitney U-test. PAP data from isolated hypoxic ventilated lungs were analysed using analysis of variance with the Student–Newman–Keuls post hoc test. *P*≤ 0.05 and *P*≤ 0.01 were considered as significant and highly significant, respectively.

## Results

### TASK-1 KO mice and expression of TASK-1 and TASK-3 mRNA in murine organs

**Fig 1:**
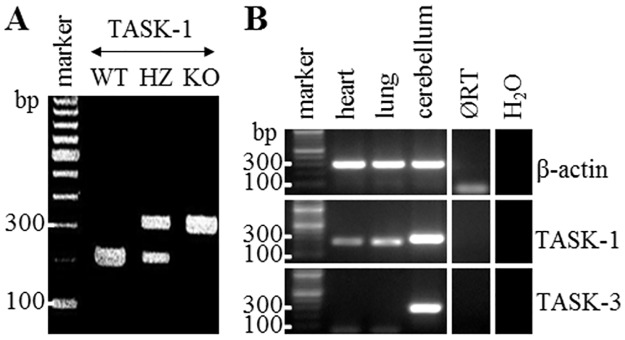
TASK-1 Knockout (KO) mice and expression of TASK-1 and TASK-3 mRNA in murine organs. **(A)** Genotype identification of TASK-1 wild-type (WT), heterozygous (HZ), and KO mice by PCR, agarose gel. **(B)** Expression of TASK-1 and TASK-3 mRNA in WT murine organs, RT-PCR, agarose gel. ØRT is lung sample processed without RT. β-Actin was used as internal control. Heart served as positive control. H_2_O is control without cDNA template.

Genotyping was performed to identify TASK-1 WT, HZ, and KO mice. A band of 234 bp and of 344 bp was detectable from WT and KO mice, respectively. In HZ animals, both bands were observed (Fig 1A). Since TASK-3 may compensate for the absence of TASK-1 [21, 22], we evaluated expression of TASK-3 mRNA along with TASK-1 mRNA in the heart, lung, and cerebellum of WT mice by RT-PCR. TASK-1 mRNA was detectable in all organs. Based on the intensity of the amplicon bands, TASK-3 was strongly expressed in the cerebellum and was absent in the heart and lung (Fig 1B).

**Fig 2:**
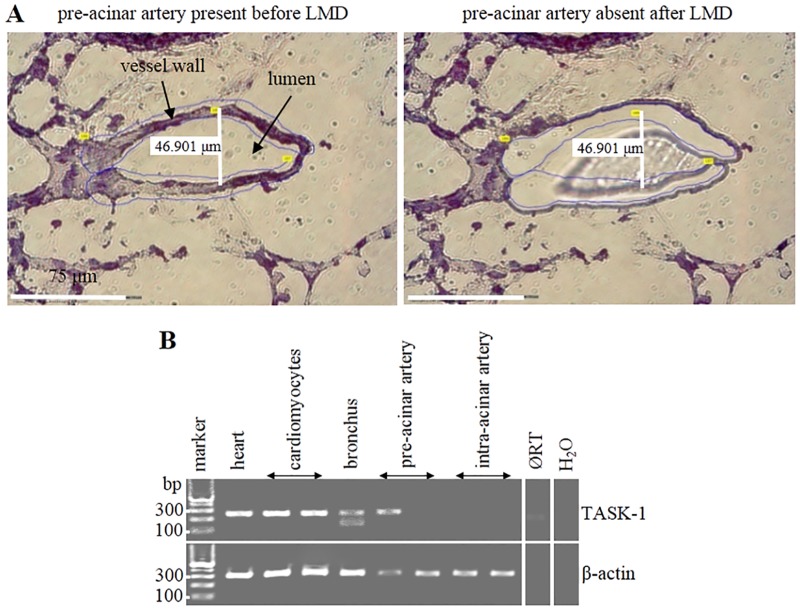
Laser-assisted Microdissection (LMD) and expression of TASK-1 in WT murine intra-pulmonary arteries. **(A)** LMD. The white line indicates the luminal diameter of a pre-acinar artery. It represents the maximum distance between intimal surfaces at right angle (90°) to the longitudinal axis. The area of vessel to be picked was first marked (blue lines; left panel). The marked piece of tissue (missing in right panel) was catapulted into the lid of a reaction tube and processed for RT-PCR. **(B)** RT-PCR of laser-assisted microdissected samples, agarose gel. ØRT is sample of cardiomyocytes processed without Reverse Transcriptase (RT). β-Actin was used as internal control. Heart served as positive control. H_2_O is control without cDNA template.

Laser-assisted microdissection (Fig 2A) combined with RT-PCR was performed to clearly define the expression of TASK-1 mRNA in pulmonary compartments of WT mice. Expression of TASK-1 was detectable in bronchi and 1 out of 2 samples of pre-acinar arteries. In intra-acinar arteries, TASK-1 was either absent or too low to be visible as a band on agarose gel. TASK-1 expression was prominent in cardiomyocytes, picked as positive control for laser-assisted microdissection and subsequent transcription/amplification processes (Fig 2B).

### Localization of TASK-1 protein in the murine lung

We evaluated TASK-1 protein expression in the lung by immunohistochemistry with different anti-TASK-1 antibodies. Since a number of publications have shown that the suitability of antibodies has to be questioned [31, 32], TASK-1 KO lungs were also included in this investigation. All antibodies labelled respiratory epithelium and bronchial and vascular SMC. However, staining intensity of these cells differed among various antibodies. Antibody R-10/14 preferentially labelled epithelium. Labelling of epithelium and SMC was of comparable intensity with antibodies R-10/15 and AB5250. Lot AN 06 of APC-024 labelled epithelium and bronchial SMC but less intensive vascular SMC, and lot AN 02 of APC-024 labelled the bronchial SMC. Surprisingly, staining intensity with all antibodies was comparable between KO and WT samples (S1 Fig).

### Expression of TASK-1 protein in murine organs

**Fig 3:**
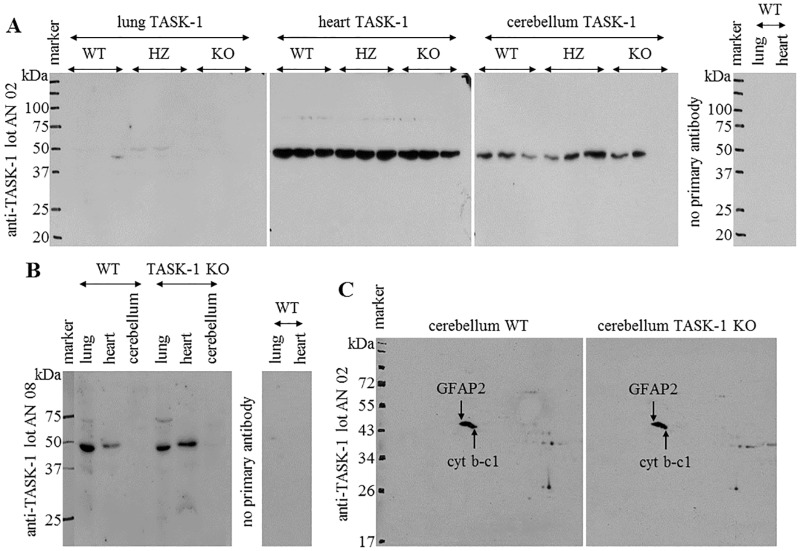
One-Dimensional (1-D) and Two-Dimensional (2-D) gel electrophoresis followed by anti-TASK-1 western blotting. **(A)** 1-D Gel electrophoresis followed by western blotting with anti-TASK-1 antibody lot AN 02. Immunoreactivity was prominent in the cerebellum and heart and was weak in lung extracts of TASK-1 wild-type (WT), heterozygous (HZ), and knockout (KO) mice. **(B)** 1-D Gel electrophoresis followed by western blotting with anti-TASK-1 antibody lot AN 08. Labelling with anti-TASK-1 antibody was detectable in lung and heart extracts of WT and KO animals, but was absent in the cerebellum of both mouse strains. Staining was absent in control without primary antibody. **(C)** 2-D Gel electrophoresis of the cerebellum of WT and TASK-1 KO mice followed by anti-TASK-1 western blotting. Comparable labelling patterns were present in samples of both mouse strains with anti-TASK-1 antibody lot AN 02. Immunoreactive spots were identified as glial fibrillary acidic protein isoform (GFAP2), and mitochondrial cytochrome b-c1 complex subunit 1 (cyt b-c1). Molecular weights (kDa) are presented on the left side of blots. The solid lines indicate assembled blots.

Western blotting performed with antibody APC-024 lot AN 02 resulted in a single band of nearly 50 kDa. For TASK-1, a molecular mass of 45 kDa can be calculated based on its amino acid sequence. Immunoreactivity of comparable intensity was observed in the cerebellum and heart of WT, HZ, and KO mice. In one out of three samples of WT lungs, a very weak band of about 47 kDa and in two out of three samples from HZ mice, a faint band of about 50 kDa was detectable. Immunoreactivity in all KO lung samples was absent or too weak to be observed on the membrane (Fig 3A). Application of lot AN 08 of the same antibody resulted in the labelling of a single band of nearly 50 kDa, too. The strongest immunoreactivity was obtained in the lung, medium in the heart, whereas cerebellum was negative. Again, no labelling difference between WT and TASK-1 KO samples was evident (Fig 3B).

2-D Gel electrophoresis combined with western blotting was performed to conclusively clarify specificity of anti-TASK-1 antibody lot AN 02 as this antibody labelled a single band of the expected size (Fig 3A). Since Medhurst et al. [33] described high expression of TASK-1 mRNA in the cerebellum and we also observed a strong signal in RT-PCR analysis (Fig 1B) and a clear labelling of a protein of the expected size was noted in western blots (Fig 3A), we used extracts of this organ for this analysis. Two immunoreactive spots were detectable on the membrane in the cerebelli of WT and KO mice (Fig 3C). The mass spectrometer-analysis identified these spots as glial fibrillary acidic protein isoform 2 (GFAP2) and mitochondrial cytochrome b-c1 complex subunit 1 (cyt b-c1). In summary, this antibody did not label the TASK-1 protein.

### Hypoxic response of intra-pulmonary arteries of WT and TASK-1 KO mice

**Fig 4:**
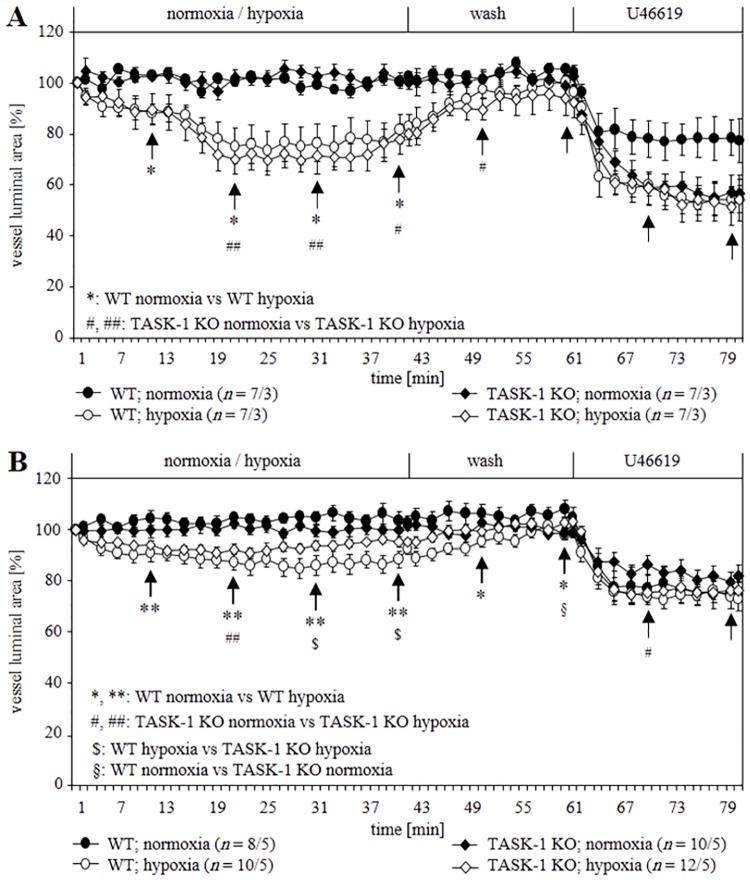
Videomorphometric analysis of the hypoxic response of intra- (A) and pre-acinar (B) arteries of WT and TASK-1 KO mice. The luminal area of vessels before exposure to hypoxia was set as 100%, and changes in the luminal areas are presented as relative values. Hypoxia-induced constriction of intra-acinar arteries was comparable in both mouse strains, whereas in KO pre-acinar arteries, it started to fade earlier as compared to WT. Data are presented as mean ± SEM. *n* is number of vessels/number of animals. Tests of significance were done at the time points indicated by arrows. *, #, §, $ P≤ 0.05 and **, ## P≤ 0.01 (Mann–Whitney U-test).

Videomorphometry was performed to evaluate the role of TASK-1 in mediating HPV in intra-pulmonary arteries. Exposure of WT intra-acinar arteries to hypoxic gassed medium (pO_2_: 40 mmHg) [24] resulted in reduction of the luminal area which reached its maximum after about 20 min (25–30% reduction of the luminal area) and started to fade after about 35 min. Control incubations with normoxic gassed medium (pO_2_: 160 mmHg) did not affect luminal area. HPV of KO intra-acinar arteries was indistinguishable from that of WT arteries (Fig 4A). HPV of KO pre-acinar arteries developed initially as in WT but faded earlier, and was significantly different from WT at 29–39 min after switching from normoxia to hypoxia (Fig 4B).

### Impact of anandamide on the reactivity of intra-pulmonary arteries

**Fig 5:**
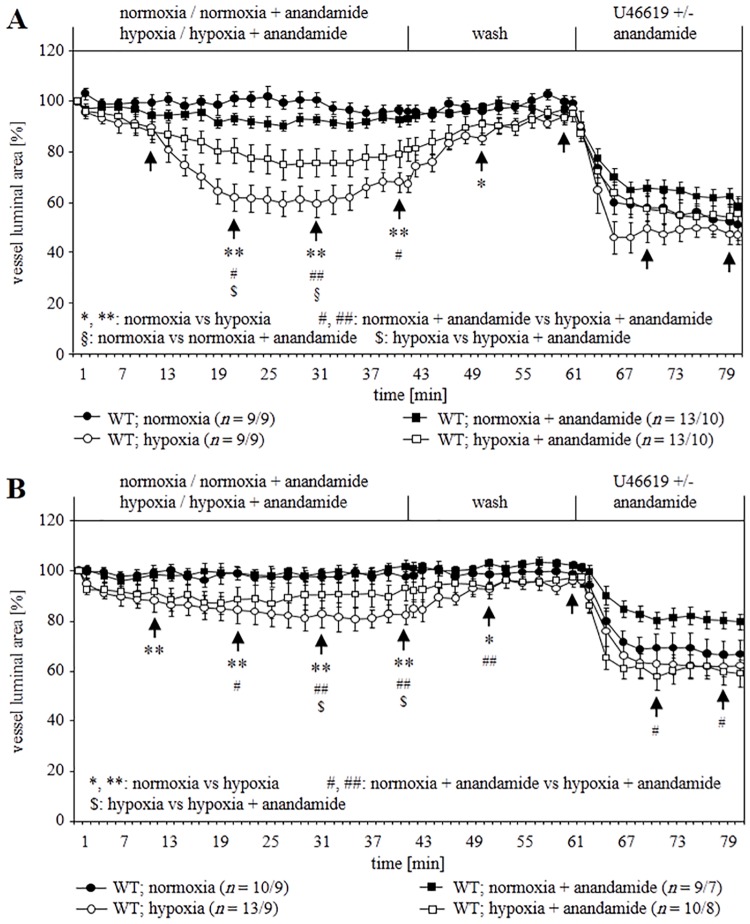
Impact of TASK-1 inhibitor anandamide on vasoconstriction of WT mice. **(A)** Effect of anandamide on intra-acinar arteries. Anandamide induced constriction at normoxia and reduced hypoxia-induced vasoconstriction significantly at one time. **(B)** Effect of anandamide on pre-acinar arteries. Anandamide did not induce constriction at normoxia. However, it reduced hypoxia-induced vasoconstriction significantly after 20 min. Data are presented as mean ± SEM. *n* is number of vessels/number of animals. Tests of significance were done at the time points indicated by arrows. *, #, §, $ P≤ 0.05 and **, ## P≤ 0.01 (Mann–Whitney U-test).

To authenticate our results from KO mice, we extended our investigations using TASK-1 inhibitors. Anandamide at submicromolar concentrations directly blocks TASK-1 [2]. In our experiments, we used an even higher concentration (10 μM) to ensure effective inhibition of TASK-1. In WT intra-acinar arteries, anandamide treatment resulted in a significant deviation from controls at only one time point each at normoxia (t = 30 min) and hypoxia (t = 20 min). This inhibitor did not prevent sustained HPV in intra-acinar arteries (Fig 5A). In pre-acinar arteries at normoxia, anandamide did not induce constriction but led to a significantly reduced HPV at 29–39 min after switching from normoxia to hypoxia (Fig 5B).

### Impact of A293 on the reactivity of intra-pulmonary arteries

**Fig 6:**
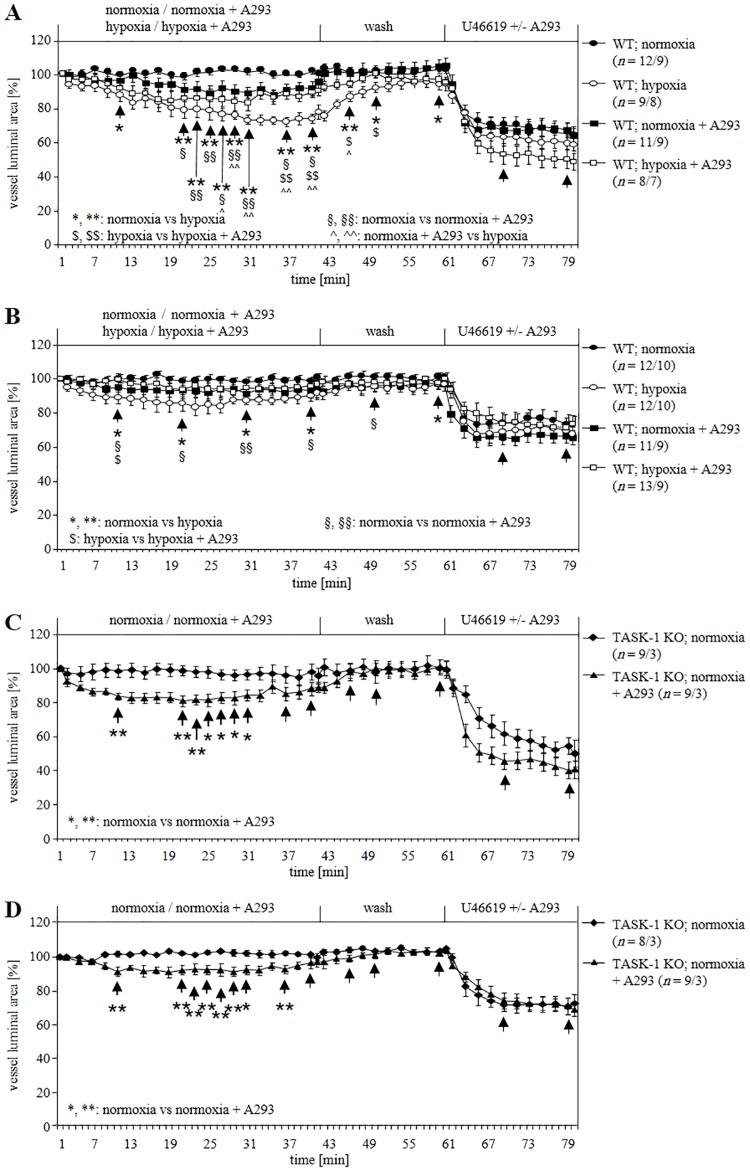
Impact of TASK-1 inhibitor A293 on vasoconstriction of WT and TASK-1 KO mice. A293 induced constriction of WT intra- **(A)** and pre-acinar **(B)** arteries at normoxia and reduced hypoxia-induced vasoconstriction. In KO intra- **(C)** and pre-acinar **(D)** arteries at normoxia, the constrictory effect of A293 persisted. Data are presented as mean ± SEM. *n* is number of vessels/number of animals. Tests of significance were done at the time points indicated by arrows. *, #, §, $ P≤ 0.05 and **, ##, §§, $ $ P≤ 0.01 (Mann–Whitney U-test).

A293 at 200 nM concentration selectively blocks TASK-1 [3, 34], thus we used the same concentration in our experiments. In WT intra-acinar arteries at normoxia, A293 caused weak but significant constriction. In hypoxic gassed medium, HPV faded after about 30 min in contrast to incubations without A293 but with DMSO (Fig 6A). Similarly, in pre-acinar arteries at normoxia, the drug also caused a significant constriction and it reduced HPV in hypoxic gassed medium (Fig 6B). This response was in accord with our hypothesis as A293 might have induced vasoconstriction while blocking TASK-1 channels. To elucidate further, A293 was administered to TASK-1 KO vessels. A293-induced constriction remained unimpaired in KO intra- (Fig 6C) and pre-acinar (Fig 6D) arteries. These data implicate that vasoconstriction induced by A293 does not operate through TASK-1.

### Acute and sustained HPV in TASK-1 KO mice and in WT mice in the presence of A293

**Fig 7:**
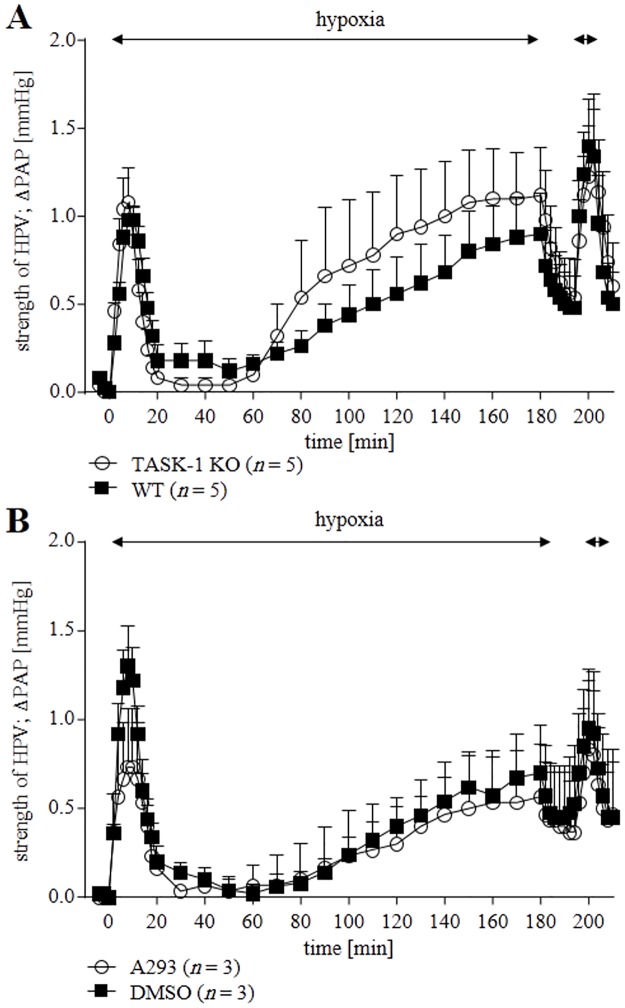
Hypoxia-induced changes in Pulmonary Arterial Pressure (ΔPAP) of isolated, perfused, and ventilated lungs of WT and TASK-1 KO mice. ΔPAP was referenced to the onset of 180 min of hypoxic ventilation period (time set at 0). Normoxic PAP and its increase during hypoxic ventilation were neither different between KO and in WT mice **(A)** nor when comparing WT mice in the presence or absence of the TASK-1 inhibitor A293 **(B)**. Data are presented as mean ± SEM. *n* refers to number of animals used. HPV: hypoxic pulmonary vasoconstriction.

We evaluated rise in PAP in hypoxic ventilated lungs from WT and TASK-1 KO mice. 180 min of hypoxic ventilation resulted in a biphasic vasoconstrictory response; in the first phase, PAP reached a maximum within 10 min followed by a pressure nadir and a second increase starting after approximately 60 min of hypoxic ventilation. A subsequent 10 min hypoxic ventilation again provoked an increase in PAP. The rise in PAP during both phases remained unimpaired in KO lungs (Fig 7A). A293 did not impair hypoxia-provoked pressure response in WT mice (Fig 7B). Thus, these results support our videomorphometry data.

## Discussion

We investigated the functional consequences of TASK-1 deficiency and inhibition on pulmonary vascular reactions, which are sensitive to hypoxia in the mouse. These include rise in PAP in isolated perfused lungs and constriction of intra- and pre-acinar arteries [24–26]. The impact of TASK-1 gene deficiency on these hypoxic responses was rather modest in that hypoxic PAP response and intra-acinar HPV were unaffected, while only maintenance of HPV for more than 30 min was impaired in pre-acinar arteries. Similarly, acute inhibition of TASK-1 by anandamide allowed for initial development of HPV in WT pre-acinar vessels but resulted in fading of HPV after 30 min. The slight but significant difference between intra- and pre-acinar arteries correlated with TASK-1 mRNA expression which was detected only in pre- but not in intra-acinar arteries. Attempts to validate mRNA expression data by immunolabelling revealed ambiguous results since, in agreement with previous findings [21, 35, 36], all available antibodies produced staining also in TASK-1 KO tissues. 2-D Gel electrophoresis combined with mass spectrometry identified the immunoreactive protein as being unrelated to TASK-1.

Vascular reactivity to hypoxia and other stimuli varies from the pulmonary trunk to the intra-acinar alveolar supply [37, 38]. We have previously shown that genetic heterozygosity for the SDHD subunit of mitochondrial complex II abolished HPV in murine intra-acinar arteries while leaving pre-acinar HPV unaffected [28]. The present data showing differential TASK-1 expression and functional consequence of its genetic deletion and inhibition in intra- versus pre-acinar arteries further underline the functional heterogeneity of these closely related vascular segments.

In the SDHD^+/-^ model affecting the intra-acinar segment, perfusion-to-ventilation matching was impaired but hypoxia-induced rise in overall PAP recorded from isolated perfused mouse lungs was unaffected. Thus, PAP is probably determined by more proximal vasoconstriction [28]. TASK-1 is expressed in more proximal vessels, including pre-acinar arteries (this study) and first to third order murine intra-pulmonary vessels [23], but its gene deficiency had no impact on hypoxic rise in PAP. In line with this observation, the constrictory responses of second order branches to vasoactive agents and hypoxia are not altered in TASK-1/3 KO mice [23]. The effect of TASK-1 deficiency or inhibition upon the sustained phase of HPV recorded from pre-acinar arteries might not have been translated into altered PAP because of its moderate extent or due to additional mechanism operating in perfused vessels (PAP recordings) compared to non-perfused vessels (videomorphometric recordings).

As TASK-1 is not critically involved in mediating HPV of intra-pulmonary arteries, one can speculate that TASK-3 might have compensated for its absence [21, 22]. Our RT-PCR analysis exhibited expression of TASK-1 but not of TASK-3 transcripts in the lungs, which is in accord with a previous report [23] where robust expression of TASK-1 mRNA but not of TASK-3 mRNA was demonstrated in murine pulmonary arteries. Thus, we can argue that TASK-3 because of its absence or very low expression in the lungs does not compensate for the absence of TASK-1 at least at this site. Recently, Pandit and coworkers [39] have presented evidence of involvement of another two-pore domain potassium channel, TASK-2 (KCNK6) [40], in the pathogenesis of PH in mice. Deficiency of KCNK6 channel in mice results in pulmonary vascular remodeling and development of PH between 8 and 20 weeks of age. Secondary branches of the pulmonary artery from 20 week old TASK-2 KO mice exhibited a greater contractile response to the thromboxane A2 mimetic, U46619. Thus, it may be speculated that TASK-1 may be functionally replaced by TASK-2 in the murine pulmonary circulation, and that TASK-2 downregulation may underlie development of PH in mice [39].

In conclusion, this study provides strong evidence that KCNK3 (TASK-1) (a) does not play a key role in initiating HPV of murine intra-pulmonary arteries, (b) may participate in maintaining HPV in murine pre-acinar arteries, but (c) does not affect hypoxia-provoked rise in PAP in the mouse. While there is compelling evidence that KCNK3 is involved in pathogenesis of PAH in humans [13–15], the mouse does not appear to serve as a suitable model for studying the underlying molecular mechanisms.

## Supporting information

S1 Fig Anti-TASK-1 immunohistochemistry of the lungs of WT and TASK-1 KO mice. Staining of smooth muscle cells (SMC; indicated by thin arrows) of vessels (marked with *) and bronchi (marked with ☖) and bronchial epithelium (indicated by arrow heads) was weak with anti-TASK-1 antibodies R-10/14 and R-10/15. Staining was prominent in bronchial epithelium and weak in SMC using AB5250 and lot AN 02. Lot AN 06 labelled both SMC and epithelium. Anti-α-smooth muscle actin stained SMC. Staining was comparable between WT and KO samples. Labelling was absent in control without primary antibody.
(TIF)

S2 Fig Cross-sectioned intra-pulmonary arteries in PCLS used for videomorphometric analysis of hypoxic pulmonary vasoconstriction. In the upper row an example of an intra-acinar and in the lower row of a pre-acinar artery is given. In the phase contrast images on the left side, the inner diameters of the vessels are given. Changes in the luminal area were analysed to quantify the hypoxic response. For this purpose, the luminal area was outlined by hand with a blue line. The pictures in the center and on the right side of the rows show the situation at normoxia and after 20 min of hypoxia, respectively. In intra-acinar artery, at hypoxia, the luminal area is reduced to 73% as compared to normoxia. The hypoxia-induced reduction of the area of the intra-acinar vessel to 85% is barely visible to the naked eye.
(TIF)
