# Supplementary figures and images for: TASK-1 potassium channel is not critically involved in mediating hypoxic pulmonary vasoconstriction of murine intra-pulmonary arteries
Source: PLoS One. 2017 Mar 16;12(3):e0174071. doi: 10.1371/journal.pone.0174071 (PMC5354433; doi:10.1371/journal.pone.0174071)

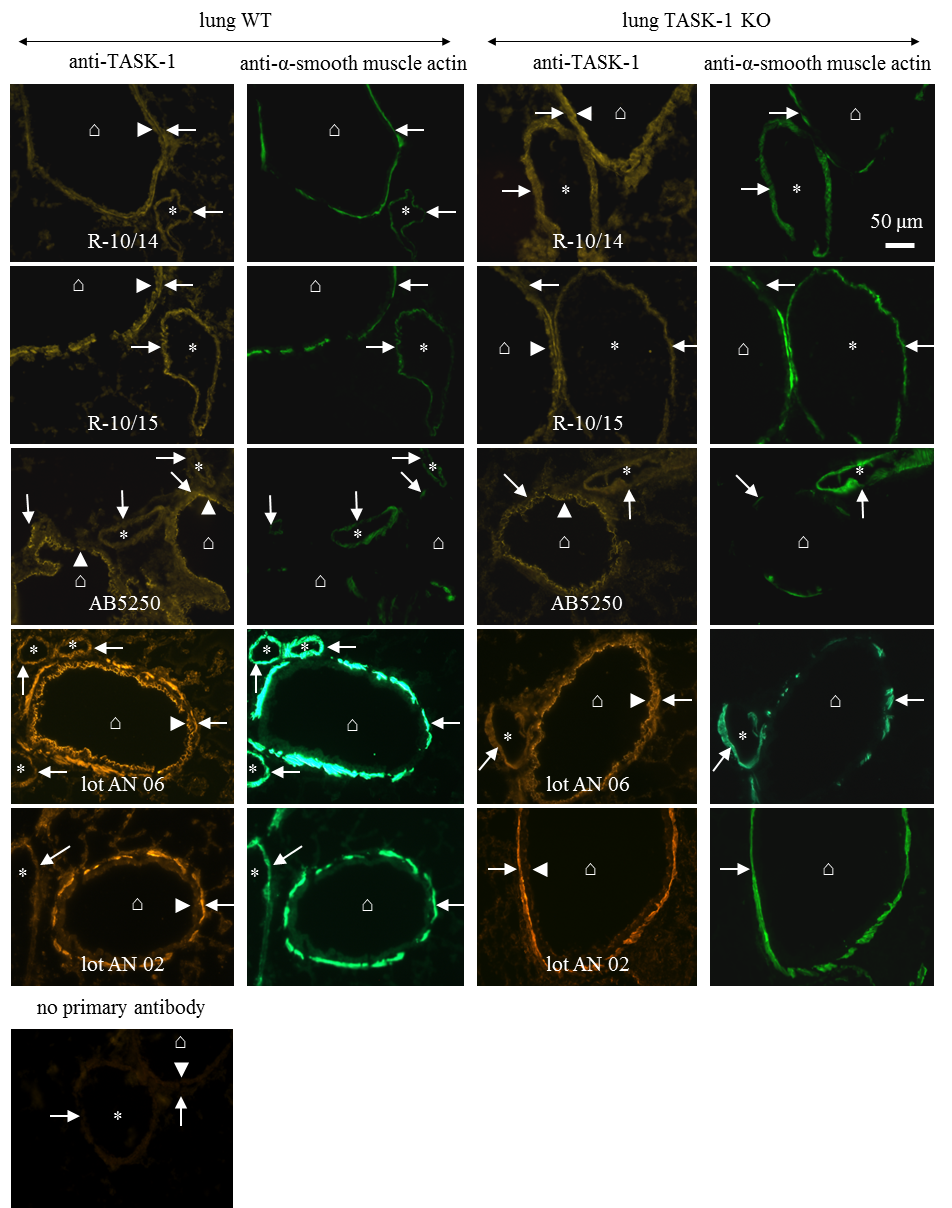

Supplement: S1 Fig — Staining of smooth muscle cells (SMC; indicated by thin arrows) of vessels (marked with *) and bronchi (marked with ☖) and bronchial epithelium (indicated by arrow heads) was weak with anti-TASK-1 antibodies R-10/14 and R-10/15. Staining was prominent in bronchial epithelium and weak in SMC using AB5250 and lot AN 02. Lot AN 06 labelled both SMC and epithelium. Anti-α-smooth muscle actin stained SMC. Staining was comparable between WT and KO samples. Labelling was absent in control without primary antibody. (TIF) [file pone.0174071.s001.tif]

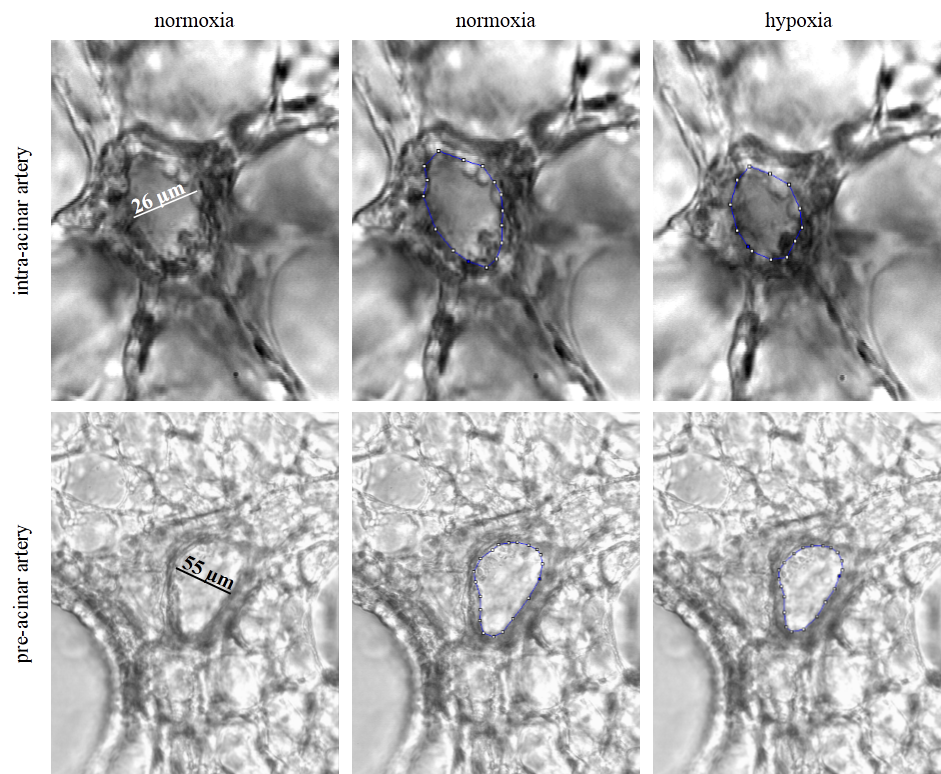

Supplement: S2 Fig — In the upper row an example of an intra-acinar and in the lower row of a pre-acinar artery is given. In the phase contrast images on the left side, the inner diameters of the vessels are given. Changes in the luminal area were analysed to quantify the hypoxic response. For this purpose, the luminal area was outlined by hand with a blue line. The pictures in the center and on the right side of the rows show the situation at normoxia and after 20 min of hypoxia, respectively. In intra-acinar artery, at hypoxia, the luminal area is reduced to 73% as compared to normoxia. The hypoxia-induced reduction of the area of the intra-acinar vessel to 85% is barely visible to the naked eye. (TIF) [file pone.0174071.s002.tif]
